# Analysis of Microbial Communities Associated with Drought-Resistant Plants Using 16S rRNA Sequencing

**DOI:** 10.3390/microorganisms14102273

**Published:** 2026-10-06

**Authors:** Irina Lyapina, Igor Fesenko, Elena Chizhevskaya, Margarita Khudyaeva, Nina Lapenko, Igor Tikhonovich, Vladimir K. Chebotar

**Affiliations:** 1 Shemyakin-Ovchinnikov Institute of Bioorganic Chemistry, Miklukho-Maklaya 16/10, Moscow 117997, Russia; fesigor@gmail.com; 2 All-Russia Research Institute for Agricultural Microbiology, Podbelskogo Hwy, 3, Pushkin, St. Petersburg 196608, Russia; chizhewskaya@arriam.ru (E.C.); mv.khudyaeva@arriam.ru (M.K.); i.tikhonovich@arriam.ru (I.T.); vladchebotar@arriam.ru (V.K.C.); 3 North Caucasus Federal Scientific Agrarian Center, Federal State Budgetary Scientific Institution, Stavropol Territory, Nikonova Str., 49, Shpakovsky District, Mikhailovsk 356241, Russia; sniish_stepi@mail.ru

**Keywords:** drought-resistant plants, microbiome, plant growth-promoting bacteria

## Abstract

Microbial communities associated with plants influence their growth, physiological functions, and resistance to pathogens and environmental stresses. Nevertheless, the identification of beneficial bacterial taxa that support plant resilience to environmental stresses and can be incorporated into synthetic microbial communities is still far from complete. To address this problem, we analyzed the microbial communities associated with three drought-resistant plant species, *Chenopodium album* L. (from wetland and arid locations), *Agropyron desertorum* (Fisch. ex Link) Schult., and *Alhagi pseudalhagi* (Bieb.) Fisch., focusing on microbial signatures in the rhizosphere, roots, and stems. We showed that even under normal conditions, the microbiomes of drought-resistant plants are enriched with bacterial taxa associated with drought resistance, such as *Bacillus* (Bacillota), Burkholderiales and *Pseudomonas* (Pseudomonadota), as well as several representatives of Actinomycetota. The greatest diversity within the endospheric microbiome was found in stem samples of *C. album* from arid location. The specific bacterial genera identified in this study, such as *Bacillus*, *Pseudomonas*, *Paenibacillus*, and *Streptomyces*, could serve as sources for the creation of inoculants for other plants to help them overcome drought.

## 1. Introduction

Drought conditions negatively impact plant growth and development. In response, plants have evolved diverse adaptive mechanisms that vary considerably among different plant lineages. The response of plants to drought can be divided into drought avoidance, drought tolerance, drought escape, and drought recovery [1,2].

One of the key factors in plant resistance to stress, particularly drought, is the beneficial microorganisms that inhabit the microbiomes of plants [3]. Recent studies have shown a link between drought tolerance and the root and rhizosphere microbiome compositions [4]. The microbial communities that inhabit the plant rhizosphere, phyllosphere (leaf and aerial parts), and endosphere include bacteria, archaea, and fungi. Some of them have been shown to increase resilience against pathogens and various abiotic stresses [5,6]. For example, plant microbiota can produce phytohormones, such as abscisic acid (ABA) and indole-3-acetic acid (IAA), 1-aminocyclopropane-l-carboxylic acid (ACC) deaminase and some volatile organic compounds (VOCs) that help plants regulate their response to abiotic stress [7,8,9,10]. In addition, rhizosphere bacteria can both reduce plant stress signaling and induce the expression of stress-related genes [11,12]. Specific microbial species are known for their ability to stimulate root development by promoting lateral root growth or enhancing root hair formation, thereby increasing a plant’s capacity to access deeper soil layers for water [13,14,15]. It has been proposed that plant growth-promoting bacteria (PGPB) promote the growth of plants and increase the root surface area or general root architecture; better roots then release higher amounts of C in root exudates, increasing microbial activity, and this cycle of events increases the amount of N available for plant uptake [16]. Furthermore, the production of osmoprotectants by rhizospheric microorganisms is considered another strategy to improve drought tolerance [17]. Additionally, bacteria infiltrating the endo- and phyllosphere are capable of promoting plant growth under stressful conditions [9]. However, despite considerable advancements in modern sequencing technologies for elucidating the composition of plant microbiomes, their functional roles in enhancing plant growth and resilience under stress conditions remain inadequately explored [18].

Under drought conditions, edaphic factors and the host genotype act synergistically: physicochemical changes in the soil serve as the primary trigger for microbiome restructuring, while the plant orchestrates its fine-tuned functional adaptation [19,20]. A drop in osmotic potential and soil desiccation sharply limit nutrient mobility, triggering a shift in the microbial community toward the dominance of oligotrophic and Gram-positive taxa (predominantly Actinomycetota) [19]. In response to this, the host genotype activates a specific transcriptional response, altering the root exudation profile (specifically by increasing the secretion of organic acids, osmolytes, and signaling phenolics). Such targeted metabolite secretion enables the plant to selectively recruit drought-tolerant strains of bacteria and fungi from the depleted soil pool that stimulate root growth and minimize osmotic stress in the host organism [20].

Previous studies have indicated that the microbiomes of drought-tolerant plants are enriched in bacterial taxa such as *Pseudomonas*, *Bacillus*, *Streptomyces*, and *Leifsonia,* which are associated with drought stress adaptation and harbor specific genes that promote plant growth and mitigate drought stress [21,22,23]. Under drought stress, the relative abundances of Actinomycetota and Bacillota (formerly Firmicutes) increase in the rhizosphere, potentially enhancing plant water-use efficiency through the production of phytohormones and exopolysaccharides (EPSs) [24,25,26]. Furthermore, research suggests that the phylum Pseudomonadota (formerly Proteobacteria) mediates systemic stress responses in plants by modulating the plant’s hormonal balance and defense signaling pathways [27,28]. Specifically, the endophytic proteobacterium *Burkholderia* was shown to significantly improve plant tolerance to both biotic and abiotic stresses, including drought [29]. In saline environments, which are closely related to drought, enrichment of Bacteroidota (formerly Bacteroidetes) has been observed, possibly contributing to plant salt tolerance through the degradation of complex organic matter and nutrient cycling [30]. Notably, Cyanobacteriophyta (formerly Cyanobacteriota) have been shown to contribute to nitrogen fixation under nitrogen-limited conditions [31]. Changes in microbial community composition under stress conditions, particularly within these key phyla, highlight the complex communication networks involved in plant adaptation. Deciphering these intricate interactions is essential for developing microbiome-based strategies to increase plant resilience in the face of environmental challenges. To date, the number of studies dedicated to analyzing the microbiome composition of drought-resistant plants is critically low. Furthermore, there are references in the literature on the composition of the microbiomes of major crops, but very little is said about the influence of the individual microbial phyla that comprise them under different conditions.

Empirical data suggest that the external application of microbial inoculants impacts not only the plants themselves but also their associated microbiomes, stimulating growth through mechanisms such as hormone production, nutrient availability, and pathogen protection [32]. Investigating the taxonomic composition of microbiomes in stress-resilient plants, particularly those exposed to drought conditions, is a critical step in designing synthetic microbial communities (SynCom) aimed at improving plant productivity [33,34]. Thus, the unique and diverse microbial communities associated with each plant species, particularly in the rhizosphere, highlight the complexity of plant-microbe interactions. Understanding these interactions further can aid in the development of microbial inoculants and biological control strategies to increase plant resilience and sustainability in agroecosystems. Continued research into the functional capacities of these microbes will facilitate the use of plant microbiomes for improved crop yields and ecological balance. Currently, there is a lack of information in the literature regarding the compositional patterns of microbiomes in plants of different genotypes grown under various conditions—particularly under drought stress—to establish a solid foundation for such research.

In this study, we aimed to characterize the microbial diversity in the rhizosphere soil, roots and stems of the following plant species: *Chenopodium album* L., *Agropyron desertorum* (Fisch. ex Link) Schult., and *Alhagi pseudalhagi* (Bieb.) Fisch. The patterns in microbial communities across plant species and tissue types were analyzed to select candidate microbial sources for enhancing plant resistance. The selected plants are drought-resistant species native to the arid regions of the Stavropol Territory (Russia) [35]. Neftekumsky and Levokumsky districts in Stavropol Territory belong to an extremely arid zone and occupy landscapes of semi-desert steppes. The climate of the area is characterized as extremely continental, with an average annual precipitation of 384 mm.

*Agropyron desertorum* and *Alhagi pseudalhagi* are drought-resistant plants that grow in the Stavropol Territory only in the extremely arid zone [35]. *A. desertorum* is notable for its cold hardiness. It tolerates low temperatures well but cannot withstand prolonged inundation (a fact that indirectly confirms its adaptation to dry rather than waterlogged conditions); this species is capable of enduring extended drought and recovers well following precipitation; it is salt-tolerant and withstands both sulfate and chloride salinity (with a moderate-to-high salt tolerance threshold of 0.2–0.6%); the stand persists for over 10 years, demonstrating high resilience to stress factors. *A. pseudalhagi* is a typical xerophyte adapted to life in arid conditions [35]. This species has developed mechanisms to adapt to moisture deficiency, such as a deep-penetrating root system, small, narrow leaves, and the regulation of stomatal activity. Moreover, *A. pseudalhagi* is capable of accumulating osmotically active substances (proline, sucrose, betaines), which help maintain the water balance of cells during dehydration. During drought, the concentration of these substances can increase tenfold. *A. pseudalhagi* can withstand significant dehydration while preserving the functionality of cellular structures. Thanks to these adaptations, the plant grows successfully in deserts and semi-deserts, where other species would not be able to survive [35]. *Chenopodium album* is considered a drought-tolerant plant. Its robust root system enables it to extract water from deep soil layers, often resulting in soil depletion. This species grows across a wide range of moisture conditions and adapts well to various moisture regimes. *C. album* is also relatively tolerant of salinity, a characteristic typical of species that survive under stressful conditions [35].

Thus, the aim of this study is to assess the microbial diversity of previously unstudied drought-tolerant species, including those capable of adapting to various conditions, as well as to compare plant and soil microbiomes and determine the influence of the host plant and the soil on their composition.

## 2. Materials and Methods

### 2.1. Plant and Soil Sample Collection

Drought-resistant plants, *Chenopodium album* L., *Agropyron desertorum* (Fisch. ex Link) Schult., and *Alhagi pseudalhagi* (Bieb.) Fisch., which grow in one spot near each other, were collected in two arid places (Neftekumsky and Levokumsky districts) in Stavropol Territory. The first place was 2 km east of Neftekumsk city, according to the site https://earthexplorer.usgs.gov/ (accessed on 23 July 2024) (44.4431° N 45.0147° E; Table S1). The soil of the site was a solonchak (meadow salt marsh). The second place was near the village of Burgun-Majary (Kurgan), Levokumsky district (44.5028° N 44.3022° E; Table S2). The soil of the site was kastanozem (FAO soil group). Samples were collected on 14 June 2023 at 30 °C and humidity at 45%. In addition, we collected samples from *Chenopodium album* L. that grew in wetland areas near the cities of Mikhailovsk and Stavropol (45.156812° N, 42.098796° E; Table S3). Samples were collected on 16 June 2023 at 24 °C and humidity at 60%. The soil of the site was a chernozem. Samples were taken at five points in each location. At each point, plants were taken together with the roots and adjacent soil. A total of five biological replicates were taken for each sample. Each plant was aseptically placed in a separate paper craft bag and immediately delivered to the laboratory. Herbarium vouchers of collected plants were deposited in the North Caucasus Federal Scientific Agrarian Center.

The plant samples were washed with tap water. Then stems, leaves and roots were separated and placed in sterile flasks with 70% ethanol solution (roots for 20 min, stems for 10 min, leaves for 5 min). The ethanol solution was then discarded, and the samples were placed in an 18% hydrogen peroxide solution (roots for 20 min, stems for 10 min, leaves for 5 min). After treatment, the samples were washed with sterile water: four times for three minutes, and then once for 20 min. The sterility check consisted of plating aliquots of the water remaining after the final wash onto plates containing GMF medium (beef enzymatic hydrolysate—15 g, NaCl—9 g, Agar—15 g, NICF, Russia). The plates were incubated at 28 °C for 2 days. The quality of surface sterilization was assessed based on the absence of microbial growth on the culture medium.

### 2.2. DNA Extraction and 16S rRNA Library Preparation

The plant genomic DNA was isolated using the DNeasy Plant Pro Kit (Qiagen, Valencia, CA, USA). DNA from the soils was isolated using the RIAM method [36].

Library preparation (60 libraries in total) for sequencing involved amplification of the target fragment of the 16S rRNA V4 variable region using universal primers (515F GTGCCAGCMGCCGCGGTAA–806R GGACTACVSGGGTATCTAAT [37]) together with linkers and unique barcodes. PCR was performed on a T100 thermal cycler (Bio-Rad Laboratories, Heracles, CA, USA) in a 15 µL reaction mixture containing 0.5 units of Q5 DNA polymerase, 5 pM of each primer, 2 mM dNTPs (Life Technologies, Carlsbad, CA, USA) and 1–5 ng of the DNA matrix. The PCR program included a denaturation step at 94 °C for 1 min, amplification of the product for 35 cycles (94 °C for 30 s, 50 °C for 30 s, 72 °C for 30 s) and final elongation at 72 °C for 3 min. An aliquot of water from the final wash of plant samples was used as a control in PCR with universal primers targeting a fragment of the 16S rRNA gene. Sterile water served as the absolute negative control.

Further sample preparation and sequencing were performed according to the Illumina protocol (“16S Metagenomic Sequencing Library Preparation”) on an Illumina MiSeq device (Illumina Inc., San Diego, CA, USA) using the MiSeq v3 reagent kit (600 cycles) with paired-end reading (2 × 300 b) (Illumina Inc., San Diego, CA, USA).

### 2.3. Bioinformatic Analysis

The raw 16S rRNA sequencing data were analyzed via the Quantitative Insights into Microbial Ecology (QIIME) pipeline v. 2020.8.0 [38]. First, the quality of the data was assessed via FastQC v. 0.12.1 and MultiQC v. 1.25.2 [39,40]. Then, the primers were trimmed from the sequences with the built-in cutadapt v. 2.10 program [41]. Dada2 v. 2020.8.0 was used for the denoising of reads [42]. The minimal allowed Phred score was 20, and the minimal overlap of paired-end reads was 99 bp. The classification of sequence reads was performed via the QIIME feature-classifier plugin v. 2020.8.0 with the Naive Bayes method. The confidence threshold for taxonomic classification was 0.7 (70%). The classifier was trained on the V4 reference sequences extracted from the SILVA database (v. 138) (https://www.arb-silva.de/; accessed on 17 July 2024). Chloroplast and mitochondrial sequences were filtered out from the aligned amplicon sequence variants (ASVs).

Alpha and beta diversity metrics were assessed with corresponding QIIME plugins to estimate the variability in microbial composition in the examined samples. Alpha diversity (within-sample diversity) was measured using the observed number of features, faith’s phylogenetic diversity (faith PD) and the Chao1 index [43,44]. The alpha-diversity bar plots were visualized in Python v. 3.7.4 [45] via the modules seaborn v. 0.11.1 [46] and pandas v. 1.2.3 [47]. Beta diversity (between-sample diversity) was assessed using Bray-Curtis sample dissimilarity in ASV abundance profiles among samples [48]. The beta diversity was visualized as a principal coordinate analysis (PCoA) plot at https://view.qiime2.org/ (accessed on 20 July 2024).

Potential functions of the microbial communities were predicted using FAPROTAX (v1.2.12) [49], and functional abundance patterns were visualized as heatmaps using seaborn 0.11.1 [46].

### 2.4. Statistical Analysis

Pairwise differences in alpha-diversity indices among sample types were evaluated using Kruskal-Wallis tests, followed by Dunn’s post hoc test with Holm correction applied for pairwise comparisons, via Python modules scipy v. 1.5.2 [50] and scikit_posthocs v. 0.14.0 [51]; adjusted *p* < 0.05 were considered statistically significant. Differences in bacterial community structure among different sample groups were tested using a QIIME built-in pairwise PERMANOVA tool based on Bray–Curtis distances, with all default settings; *p* < 0.05 were considered statistically significant.

## 3. Results

### 3.1. Overview of Sequencing Data

**Figure 1:**
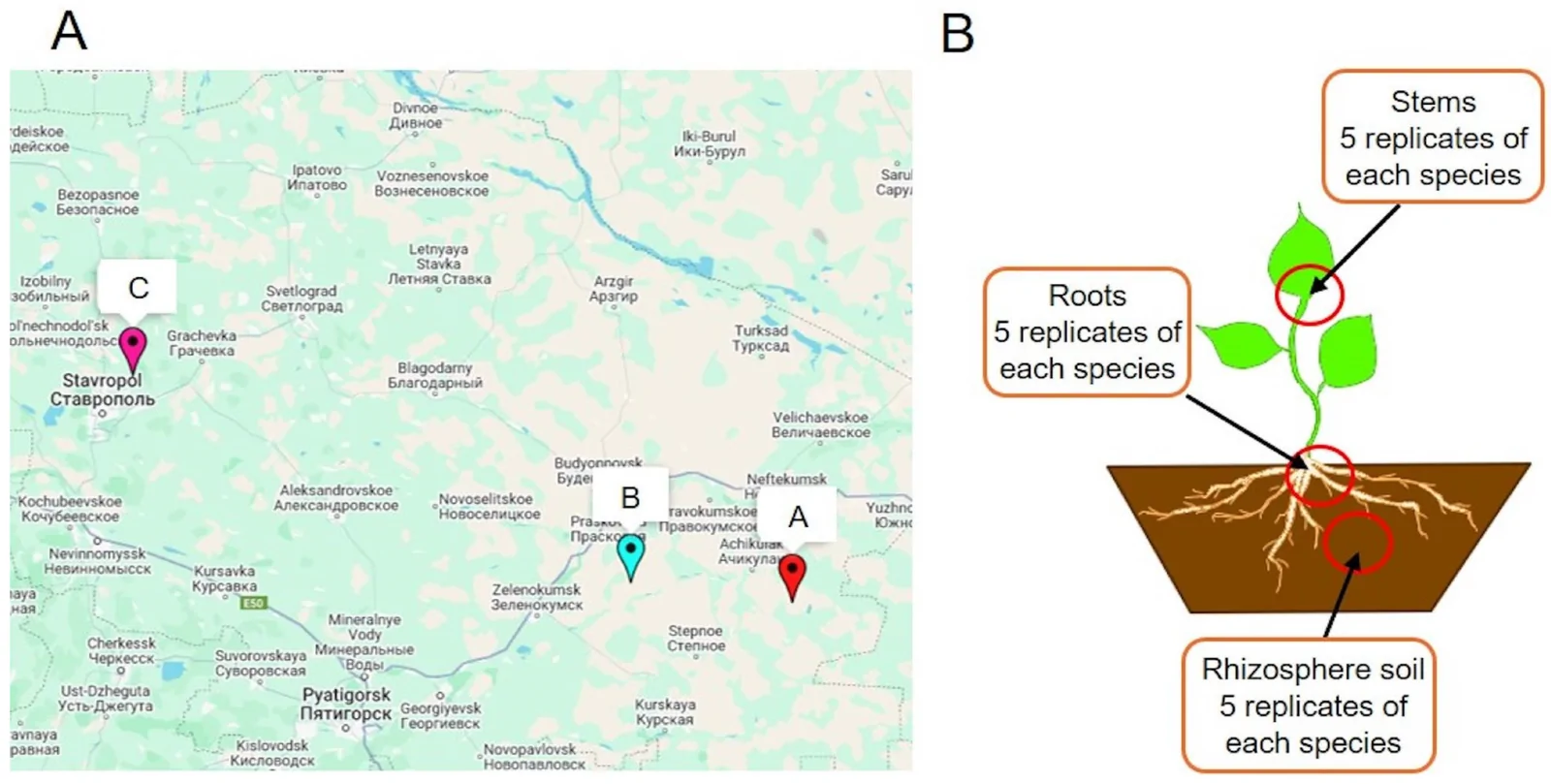
Sample collection. (**A**) Map of the sample collection sites (A—44.4431° N 45.0147° E; B—44.5028° N 44.3022° E; C—45.156812° N, 42.098796° E). The map was created at https://maps.co/ (accessed on 5 June 2025). (The Russian names are duplicated in English.) (**B**) Plant microbiome sampling scheme.

The samples of the endospheric microbiomes from stems and roots, as well as the rhizosphere microbiomes, were collected from drought-resistant plants (*Chenopodium album* L., *Agropyron desertorum* (Fisch. ex Link) Schult., and *Alhagi pseudalhagi* (Bieb.) Fisch) in the Neftekumsky district of the Stavropol Territory (Figure 1A). The main approach for collecting samples of drought-resistant plants was to search for places where all the studied plants were in one place, growing on the same soil. This made it possible to compare the microbiomes of plants and soils and determine what effect the host plant and the soil on which these plants grow have on the formation of the epiphytic and endophytic microbiome. Overall, 30 samples from arid locations and 10 samples from a wetland site, comprising five replicates of stem and root tissues of each species, were collected (Figure 1B). In addition, rhizosphere samples (five replicates from each location) were collected for analysis. Following DNA extraction, 60 16S rRNA libraries were prepared and used for sequencing.

The quality of the raw data after sequencing was checked using the FastQC and MultiQC programs [39,40]. Reads with Phred scores below 20 were discarded. The estimated length of the amplicon was 291 nt (515F-806R).

Table 1 presents the mean read counts at various filtration stages, along with the standard deviation. The higher read counts in the aerial (green) parts of the plants compared to rhizosphere samples indicated greater sequencing depth, which correlates with the greater biomass of these samples (Table 1). For the green parts (stems) of *A. pseudalhagi*, *A. desertorum*, and *C. album*, 37,500 to 50,000 reads were obtained (Table 1). The highest read count, over 60,000, was observed on the stems of *C. album* L. from wetland location (Table 1).

After quality filtering, a high proportion of reads (an average of 85.36%) were retained across all samples, ensuring reliable downstream analysis (Table 1). On average, 68% of the reads were merged, and 52.7% of the total reads were identified as non-chimeric, indicating the quality of the obtained data (Table 1). In total, we obtained 3683 amplicon sequence variants (ASVs) representing unique microbial taxa for plants from arid locations and 1731 ASVs for plants from the wetland site. The average length of 253.45 bp across all the samples indicates good sequence coverage and resolution for microbial diversity profiling.

To get a reliable picture of the dominant and abundant genera or families within samples, we analyzed the rarefaction curves. The curves reached a plateau at a sequencing depth of 7000 reads, which corresponds to a typical depth for standard microbiome studies. Thus, we normalized the depth while preserving the greatest number of features across the greatest number of samples.

### 3.2. Composition of the Microbial Communities of Drought-Resistant Plants

**Figure 2:**
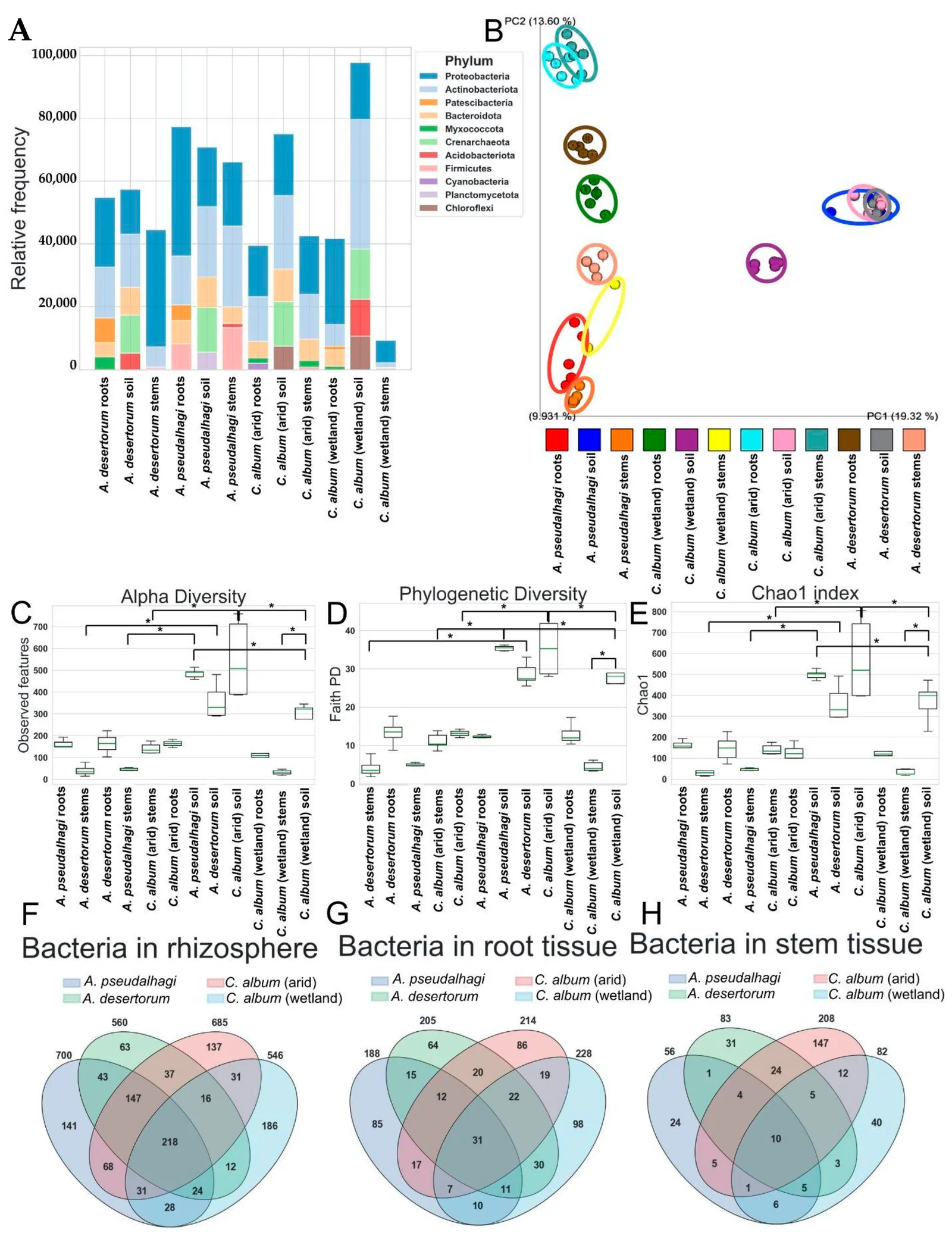
Microbial diversity of the samples. (**A**) Bar plot showing five phyla with the highest relative abundance in each sample; (**B**) PCoA of Bray–Curtis dissimilarity between samples; (**C**) observed number of features within each sample (Kruskal-Wallis with Dunn’s test: * = *p*-value < 0.05); (**D**) Faith’s phylogenetic diversity (Kruskal-Wallis with Dunn’s test: * = *p*-value < 0.05); (**E**) Chao1 index (Kruskal-Wallis with Dunn’s test: * = *p*-value < 0.05); (**F**) Venn diagrams showing the number of unique species in rhizospheric soil, (**G**)—in root tissues, and (**H**) in stem tissues.

To determine the taxonomic affiliations of the identified ASVs, we used the SILVA reference database (https://www.arb-silva.de; accessed on 17 July 2024). The detected ASVs were assigned to 35 phyla, 92 classes, 212 orders, 312 families, 540 genera, and 323 species (Figure 2A; Table S4). The most abundant phyla were presented by Pseudomonadota, Actinomycetota, Patescibacteria, Bacteroidota, Myxococcota, Thermoproteota, Acidobacteriota, Bacillota, Cyanobacteriophyta, Planctomycetota and Chloroflexota (Figure 2A). Pseudomonadota (formerly Proteobacteria), Actinomycetota (formerly Actinobacteriota), Acidobacteriota, Bacteroidota (formerly Bacteroidetes), Bacillota (formerly Firmicutes), and Verrucomicrobiota are well-known constituents of plant and soil microbiomes [52,53,54,55,56,57,58,59]. Some of the species from the phyla Bacillota, Actinomycetota and Pseudomonadota identified across all the samples are known to exhibit plant growth-promoting (PGP) abilities [52,54,55]. In addition, we found species from the Armatimonadota, Chloroflexota (formerly Chloroflexi), Planctomycetota and Patescibacteria taxa, which have been shown to thrive in conditions with various limitations, such as nutrient limitations [60,61,62,63]. In the rhizosphere, we also identified two Archaea phyla, Thermoproteota (formerly Crenarchaeota) and Thermoplasmatota, which are also known to be important components of soil microbiomes (Table S4) [64]. The consistent prevalence of such phyla as Bacteroidota (formerly Bacteroidetes), Bacillota (formerly Firmicutes), Verrucomicrobiota, and (formerly Cyanobacteriota) across different plant parts and the rhizosphere acknowledges that these microbial groups perform overlapping or similar functions, which are potentially critical for nutrient uptake, the stress response, and overall plant fitness (Table S4). Notably, the recurring appearance of Myxococcota, known for their role in biodegradation and nutrient mineralization, underscores their potential contribution to root health and plant vigor [65].

Cyanobacteriophyta were found among the most abundant phyla in root samples of *C. album* collected in arid areas, compared to samples collected from wetland areas (Figure 2A). This phylum was also highly represented in *A. desertorum* stems (Figure 2A). These data suggest that microorganisms from this phylum may contribute to the drought tolerance of *C. album*.

The functional abundance prediction based on FAPROTAX revealed distinct functional profiles for virtually all samples, yet common patterns were observed for the soil samples (Figure S1). Functional groups such as aerobic ammonia oxidation, predatory or exoparasitic, and groups involved in nitrite-related and oxidation processes were common for soil samples. Functional groups of aerobic chemoheterotrophs and chemoheterotrophs were identified in the majority of samples, with the exception of the stems. The phototrophy group was absent from *C. album* samples from wetland areas but was abundant in samples from arid areas. In contrast, the nitrogen fixation functional group was more abundant in *C. album* samples from wetland sites than in samples from arid areas (Figure S1).

The PCoA of the diversity among the samples revealed that all the rhizosphere samples from the arid location were clustered together and away from the other samples (Figure 2B). Rhizosphere samples of *C. album* from the wetland area clustered separately from all samples (Figure 2B). These data support the conclusion about the influence of environmental conditions on the formation of the soil microbiome, even within a single plant species growing under different conditions [66]. In addition, recent studies indicated that plants change the composition of the rhizosphere microbial community through root exudates [67].

The endosphere samples (roots and stems) of *C. album* from arid areas and *A. pseudalhagi* clustered together with respective species and were separated from the other (Figure 2B). Endosphere samples of *C. album* from the wetland site differed from *C. album* samples from arid area, suggesting that the microbiomes of this plant vary greatly depending on growing conditions (Figure 2B).

The PERMANOVA test results indicated that genotype, as well as affiliation with plant tissues or rhizosphere soil, has a significant effect on microbial community composition (Table S5). According to the results of this test, the differences between communities in *C. album* samples from wetland areas, as well as between *C. album* stem samples from arid and wetland areas, were statistically insignificant. The difference between soil samples from *A. pseudalhagi* and *A. desertorum* was also statistically insignificant (Table S5).

#### 3.2.1. Analysis of Microbial Diversity in the Roots and Rhizosphere

In line with previous studies, our analysis revealed the abundance of the phyla Pseudomonadota (formerly Proteobacteria), Actinomycetota (formerly Actinobacteriota), and Bacteroidota (formerly Bacteroidetes) in the root and rhizosphere microbiomes. We found a similar diversity of Pseudomonadota and Bacteroidota genera in the root samples of *C. album* when comparing arid and wetland conditions (Table S4). However, the number of Actinomycetota genera was lower in the wetland root samples in comparison to the arid area (22 vs. 36 genera; Table S4). We also found that the number of genera for Pseudomonadota, Actinomycetota, and Bacteroidota phyla from *C. album* arid rhizosphere samples was higher than in rhizosphere samples from wetland areas (70 vs. 62, 57 vs. 42, and 39 vs. 20 genera, respectively; Table S4). The diversity of Bacteroidota genera was similar in rhizosphere samples of *C. album* from arid areas, *A. pseudalhagi* and *A. desertorum*.

Among the most frequently occurring genera in root samples from arid areas were *Pantoea*, *Bacillus*, *Pseudomonas*, *Paenibacillus*, and *Streptomyces* (Table S4). Of these genera, only *Pantoea* was not represented in the root samples of *C. album* from the wetland area (Table S4). Among the most represented genera were *Ohtaekwangia* and *Acidibacter* in the root samples of *C. album* from wetland area, as well as *Microvirga*, *Rubrobacter*, *Blastococcus* and *Skermanella* in all soil samples (Table S4).

In addition, we found that in the root samples, *A. pseudalhagi*, *C. album* from wetland area, and *A. desertorum* shared the dominant phylum *Patescibacteria*, while *A. desertorum* and *C. album* from wetland and arid areas shared the dominant phylum *Myxococcota* (Figure 2A). All rhizosphere samples were distinguished by the presence of the dominant phylum Thermoproteota (formerly Crenarchaeota), which belongs to the Archaea dominion, as indicated above (Figure 2A).

The compositions of the root and rhizosphere microbiomes tend to be highly similar and share dominant phyla. Our analysis revealed that the rhizosphere harbored greater microbial diversity, which is consistent with findings from previous studies (Figure 2C–E) [68]. According to the alpha-diversity analysis, all the samples from the soil had the highest number of observed features, as did the Faith PD and Chao1 index, indicating that these samples contained the greatest diversity of microbial species (Figure 2C–E). However, in the rhizosphere samples of *C. album* from wetlands, microbial diversity was somewhat lower, although still significantly higher than that in root and stem samples (Figure 2C–E). *A. pseudalhagi* and *C. album*, both from arid and wetland areas, particularly exhibit the highest rhizosphere diversity (Figure 2F). Dunn’s post hoc tests revealed significant differences between stem and soil samples for all plant species. No significant group differences were detected for all root samples. Moreover, soil samples from *C. album* from wetland areas differed significantly from soil samples from *A. pseudalhagi* and *C. album* from arid locations (Figure 2C–E).

The root tissue samples of *C. album* from wetlands had the highest number of unique, nonintersecting species (Figure 2G). However, according to the alpha-diversity analysis, a significantly lower diversity was observed within all the samples from the roots than in those from the rhizosphere (Figure 2C–E).

#### 3.2.2. Analysis of Microbial Diversity in Phyllosphere Samples

In contrast to the rhizosphere microbiome, the endosphere microbiome is usually not particularly diverse but is characterized by high stability [69]. According to recent research, the dominant phyla in the endosphere and phyllosphere (aboveground parts) of angiosperms are Bacillota (formerly Firmicutes), Acidobacteriota, Actinomycetota (formerly Actinobacteriota), and Cyanobacteriophyta [70]. Our analysis revealed that the Actinomycetota (formerly Actinobacteriota) was one of the dominant phyla in all the samples, along with the atypical Pseudomonadota (formerly Proteobacteria) (Figure 2A).

Our analysis revealed that the diversity of genera for Pseudomonadota (formerly Proteobacteria) phylum was similar in the *A. pseudalhagi* and *A. desertorum* stem samples. The number of genera was higher in *C. album* stem samples, with *C. album* from arid areas having the highest number of genera (43 vs. 28 in wetland samples; Table S4). The highest diversity of Actinomycetota (formerly Actinobacteriota) genera was represented in *C. album* from arid area and *A. desertorum* stem samples, while a smaller number of Actinomycetota genera was found in *A. pseudalhagi* and *C. album* from wetland area (33 and 27 vs. 12 and 13, respectively; Table S4). Among the most frequently occurring genera in all stem samples from arid areas were *Arthrobacter*, *Sphingomonas*, *Massilia* and *Pseudomonas* (Table S4). All these genera, except *Arthrobacter*, were also found in *C. album* stem samples from wetland areas (Table S4). We also found that stem samples from each species had at least one prominent dominant phylum that distinguished them from the others (Figure 2A). Thus, *C. album*, both from wetland and arid regions, had Myxococcota, *A. pseudalhagi* had Acidobacteriota, and *A. desertorum* had Cyanobacteriophyta (Figure 2A).

#### 3.2.3. Comparison of Uniquely Represented Taxa in All the Samples

Next, we sought to compare the unique nonintersecting microbial species of all the samples. Unique species in the rhizosphere samples were assigned to an average of 116 genera and 95 families in *A. pseudalhagi* and *C. album* from both wetland and arid areas, as well as 55 genera and 44 families in *A. desertorum*, which belong to an average of 17 phyla.

The presence of unique genera such as *Nakamurella* and *Pantoea* in the rhizosphere microbiome of *A. pseudalhagi*, and *Oceanobacillus* in *A. desertorum,* further highlights the plant-specific microbial adaptations likely facilitating nutrient acquisition and stress mitigation in the rhizosphere (Table S4). Actinomycetota and Pseudomonadota, such as *Nakamurella* and *Pantoea,* have been shown to have plant growth-promoting properties and can also reduce abiotic stress [71,72]. Representatives of Bacillota (*Oceanobacillus*) are also known as plant growth-promoting bacteria, which likely explains its presence in the *C. album* samples from the wetland location as well [5,73]. Among the unique genera in the *C. album* wetland rhizosphere samples was *Singulisphaera* from the Planctomycetota phylum, which is specific to wetlands and was considered to be a part of the “core species” beneficial for plants [74].

The unique species in root samples were assigned to 62 genera and 52 families (for *A. pseudalhagi* and *A. desertorum*) and to 79 genera and 54 families (for *C. album* from arid area), representing an average of 11 phyla. In *C. album* root samples from the wetland location unique species were assigned to 60 genera and 59 families, belonging to 14 phyla. The presence of genera such as *Pseudomonas* in *A. pseudalhagi*, *Paenibacillus* in *A. pseudalhagi*, in *A. desertorum* and in *C. album* from wetland area, *Oceanobacillus* in *A. pseudalhagi*, *Sphingomonas* in *C. album* from arid and wetland area, *Bacillus* in *C. album* from arid area and *Ralstonia* in *A. pseudalhagi* in the root microbiomes aligns with their well-established roles in promoting plant growth through mechanisms such as nitrogen fixation and phytopathogen suppression [7,22,73,75,76,77], further substantiating the beneficial interactions occurring within the root endosphere (Table S4). In *C. album* root samples from the wetland location, the unique genus *Armatimonadales* was found, among others, which is commonly present in rhizosphere and plant parts, suggestively promoting plant growth [78,79].

The unique species in stem samples from arid locations were assigned, on average, to 26 genera and 25 families (for *A. pseudalhagi* and *A. desertorum*) and to 100 genera and 61 families for *C. album*, belonging to seven and 13 phyla, respectively. In *C. album* stem samples from the wetland site unique species were assigned to 33 genera and 34 families, belonging to seven phyla. This high number of unique genera in *C. album* samples from an arid area may indicate unique processes that contribute to the adaptation to drought conditions of a plant with a wide range of growing aerials, compared to other drought-tolerant plants examined in this study, as well as compared to *C. album* plants growing in wetland areas. The specific unique taxa identified in the stem microbiomes of the examined species included *Pseudogracilibacillus* and *Streptococcus* (Bacillota) in *A. pseudalhagi*; *Clavibacter* (Actinomycetota) and *Paenibacillus* (Bacillota) in *A. desertorum* and in *C. album* from wetland area; and *Devosia* (Pseudomonadota) and *Arthrobacter* (Actinomycetota) in *C. album* from arid area (Table S4).

## 4. Discussion

The primary aim of this study was to gain insight into the diversity of microbial communities in plants capable of surviving prolonged stress, particularly drought. To broaden the picture, we selected three representatives of drought-tolerant species characteristic of the extremely arid regions of the Stavropol Territory (Russia)—*Chenopodium album* L., *Agropyron desertorum* (Fisch. ex Link) Schult., and *Alhagi pseudalhagi* (Bieb.) Fisch. *C. album* is an unpretentious plant and grows in all soil and climatic zones of the region, including in the extremely arid zone, making it an interesting subject for research. We also analyzed samples of *C. album* from a wetland region to assess the influence of environmental conditions on the formation of microbiome communities.

According to the obtained results, the composition of each species’ endophytic microbiome clearly differed both from one another and from the rhizospheric microbiome. Furthermore, the endo- and exomicrobial composition of *C. album* from the wetland area differed significantly from all the others (Figure 2B). Taken together, these results may indicate that despite the commonality of the “resource” soil microbiome formed under the same stress conditions, each plant, depending on its genetic or physiological characteristics, recruits a certain specific pool of endogenous microorganisms [18]. The type of soil in which the plant under study grew can also introduce certain variations by shaping the initial pool of microorganisms available for plant recruitment. Nevertheless, each plant acts as a selective filter, choosing specific beneficial microbes regardless of the soil’s initial characteristics [80]. Soil type exerts the greatest influence on the rhizosphere microbiome, as demonstrated by our results, which showed high diversity in these samples [81].

The most common bacteria in the rhizosphere of the majority of angiosperms belong to the phyla Pseudomonadota (formerly Proteobacteria), Actinomycetota (formerly Actinobacteriota), and Bacteroidota (formerly Bacteroidetes) [82,83]. Our analysis also confirmed their presence in the root and rhizosphere microbiomes. Specifically, the Pseudomonadota and Actinomycetota phyla are known for their diverse metabolic capabilities, including nutrient cycling, plant growth promotion, and biocontrol activities, suggesting a foundational microbial framework supporting the host plants [84,85].

Such genera as *Pantoea*, *Bacillus*, *Pseudomonas*, *Paenibacillus*, and *Streptomyces*, identified in root samples from arid areas, are frequently associated with drought tolerance in the literature (Table S4) [7,21,22,23,86]. Among the most frequently encountered genera in *C. album* root samples from the wetland area were *Ohtaekwangia*, often found in nodules, and *Acidibacter*, the abundance of which depends on soil nitrogen levels; this finding may be potentially linked to the nitrogen-fixing capability of *C. album* described in the literature [87,88,89]. Among the most frequently encountered genera among all soil samples were *Microvirga*, *Rubrobacter*, *Blastococcus* and *Skermanella*, which are potentially associated with plant growth promotion and are capable of surviving in extreme conditions (Table S4) [90,91,92,93].

Alpha-diversity analysis showed that all rhizosphere soil samples exhibited high microbial species diversity, indicating that a robust microbiome potentially contributes to their host-plant adaptability under varying environmental conditions. Potentially, such a high number of unique species identified in *C. album* from wetland samples indicates a strong difference between the recruited microorganisms under conditions of different water availability. This observation aligns with studies showing that plants in stressful environments often recruit a more distinct microbiome to increase their resilience [94].

Since soil microbial communities are able to colonize the phyllosphere, the identification of Pseudomonadota in stem samples is easily explained by the settling of soil microorganisms [95]. Moreover, recent studies suggest that certain environmental factors, such as drought, may influence the increased abundance of soil microbial taxa with phylogenetically conserved functions in the phyllosphere compared with those in soil samples [96]. For example, the most abundant order in *A. desertorum* stem samples was Pseudomonadotal Burkholderiales, which are known as beneficial bacteria that influence drought tolerance in a wide range of plant hosts (Table S4) [97,98]. Interestingly, Burkholderiales were also among the most abundant microorganisms in stem samples of *C. album* from wetlands, along with *Pantoea*, *Pseudomonas* and *Bacillus*, which may be related to the wide distribution of *C. album* and its ability to survive in a variety of conditions (Table S4) [99].

In all stem samples from arid regions, we also identified genera for which growth-promoting properties and an association with drought tolerance have been described in various studies (*Arthrobacter*, *Sphingomonas*, *Massilia* and *Pseudomonas*) (Table S4) [23,73,100]. These same genera were also found in *C. album* stem samples from wetland areas, which may indicate their potential role in enhancing growth processes under changing conditions (Table S4).

Our results are consistent with the conclusion drawn in previous studies that the endosphere microbiome of each plant comprises a significant proportion of microorganisms unique to that plant. Moreover, the diversity within the stem samples was the lowest among all the samples (Figure 2C–E). The one that stands out most from the other stem samples is *C. album* from arid area, which presents a significantly greater number of unique nonintersecting microbial species (147) than *A. desertorum*, *A. pseudalhagi* and *C. album* from wetland area (31, 24 and 40, respectively; Figure 2H), resulting in greater diversity within the sample (Figure 2C–E). This difference may be related to physiological differences in *C. album* compared to other plants from the arid region, as well as the need to adapt to stressful conditions compared to *C. album* plants growing in normal humidity conditions. It is known from the literature that *C. album* also possesses more living cells, whereas other species exhibit a more xeromorphic structure, which may contribute to the greater diversity of observed features [35,99].

*C. album* harbors distinct features compared to other examined species from the arid region. This is expressed, among other things, by a more developed architecture of leaves and stems, which respond to drought by reducing their number in order to conserve water [99]. Due to this, as well as its deeper-penetrating roots, *C. album*, unlike the *A. desertorum* and *A. pseudalhagi*, is more widespread and can grow both in fairly arid areas and in areas with normal moisture. Differences between *C. album* plants from arid regions and those from wetland areas likely lead to recruiting significantly different microbiomes for adaptation under stress conditions. In addition, *C. album* possesses a high content of proline, known for its osmoprotective properties, and a high capacity for nitrogen absorption, which promotes plant growth [101]. All these characteristics, which distinguish *C. album* from other drought-tolerant plants studied, may account for the higher microbiome diversity in samples of this species, particularly under arid conditions, thereby facilitating plant adaptation to a wider geographical range.

The unique, species-specific stem microbes identified in this study likely play a role in conferring stress resistance to the plant’s aerial parts, mobilizing nutrients, or even protecting against leaf-attacking pathogens, although further research is needed to elucidate their functions. Our findings also support the reported conclusion that the phyla Bacillota, Acidobacteriota, and Actinomycetota are commonly found in the stem tissue microbiome [102]. Interestingly, the unique genus *Deinococcus* was detected in *C. album* stem samples collected from a wetland site; it is typically associated with the rhizosphere microbiome and is considered beneficial due to its antioxidant properties and role in nutrient cycling, according to the literature [103].

Considering recent findings concerning the role of the plant microbiome in stress response and adaptation [104], the unique microbial diversity observed in the rhizosphere and endosphere indicates a complex interplay between the plants and their microbial associates. Such diversity can enhance the resilience of these plants to biotic and abiotic stressors, underscoring the importance of preserving microbial diversity as a component of sustainable agricultural practices. Further research to identify the composition of these microbial communities at deeper resolution is an interesting goal for the near future.

Although 16S rRNA sequencing is typically considered limited, it is a common tool for identifying general microbial communities’ patterns. Furthermore, shotgun metagenomics requires a larger sample biomass and a greater quantity of extracted DNA. Undoubtedly, the metagenomic analysis of the selected plants is of great interest to us, given the species-level resolution and the ability to directly identify the functional affiliation of the microorganisms. However, the aim of this study was to assess the microbial diversity of previously unstudied drought-tolerant species, including those capable of adapting to various conditions, and to identify areas of interest for further research.

## 5. Conclusions

This study provides valuable insights into the composition and diversity of the microbiomes associated with three drought-resistant plant species. The observed differences in microbial communities across plant species and tissues suggest that specialized interactions are driven by plant genotype and environmental pressures. The presence of unique taxa with predicted functional roles in nutrient cycling, stress tolerance, and plant growth promotion underscores the importance of the microbiome in the adaptation and resilience of these plants in arid environments.

The mechanism by which plants are able to recruit desired microorganisms into their microbiome based on their own needs is very poorly understood. In recent years, this topic has been increasingly explored in studies such as those of Ait-El-Mokhtar et al. [105] and Nataraja et al. [106], but it still remains incompletely understood. Thus, further in-depth study of this topic is required, using a variety of plants, specifically both drought-tolerant and drought-sensitive ones, as well as subjecting them to various stress conditions, to confidently link specific types of microorganisms to the effects they exert on different plants.

## Figures and Tables

**Table 1:** 16S rRNA sequencing statistics.

Sample	Total Reads (SD)	Quality Filter (SD)	Merged Reads (SD)	Non-Chimeric Reads (SD)	% of Non-Chimeric Reads (SD)	Sampling Site
*A. pseudalhagi* soil	33,738 (1746.3)	30,142 (1673.4)	20,009 (1217.8)	19,226.2 (1110.8)	56.9 (1.5)	Neftekumsk, Burgun-Majary (Kurgan)
*A. desertorum* soil	29,740.8 (4557.8)	26,554.4 (4169.5)	16,129 (3752.1)	15,443 (3664.5)	51.4 (4.1)	Neftekumsk, Burgun-Majary (Kurgan)
*C. album* (arid) soil	36,020.4 (2839.3)	31,793 (2605.3)	21,766.4 (3151.2)	21,156.6 (3616.1)	58.4 (5.6)	Neftekumsk, Burgun-Majary (Kurgan)
*C. album* (wetland) soil	55,840.2 (19,144.2)	45,886.4 (15,934.03)	26,865 (10,004.02)	23,922.4 (8078.8)	42.82 (2.3)	Mikhailovsk and Stavropol
*A. pseudalhagi* roots	43,136 (8304.8)	38,748.4 (7475.6)	31,755.4 (6802.3)	24,617.8 (5553.2)	56.99 (5.1)	Neftekumsk, Burgun-Majary (Kurgan)
*A. desertorum* roots	34,104 (9503.7)	30,475.4 (8695.6)	22,161.6 (6405)	15,301.8 (4922.4)	44.4 (2.8)	Neftekumsk, Burgun-Majary (Kurgan)
*C. album* (arid) roots	35,951 (9002.1)	32,309.8 (8223.4)	24,767.2 (7173.4)	19,831.8 (4969.9)	55.3 (3.9)	Neftekumsk, Burgun-Majary (Kurgan)
*C. album* (wetland) roots	57,501.8 (12,549.8)	46,827 (9939.8)	34,554.6 (7924.3)	25,765.4 (5332.6)	44.93 (2.4)	Mikhailovsk and Stavropol
*A. pseudalhagi* stems	49,857.6 (4303.5)	44,819.8 (3895.8)	41,412 (3498.1)	30,887.2 (2499.5)	62 (1.5)	Neftekumsk, Burgun-Majary (Kurgan)
*A. desertorum* stems	40,814.8 (8606.7)	36,466.4 (7683)	33,020.6 (6842.1)	26,152 (4959.4)	64.3 (1.6)	Neftekumsk, Burgun-Majary (Kurgan)
*C. album* (arid) stems	37,690.6 (3108.4)	33,850.2 (2823.2)	25,651 (2677.1)	21,392.2 (1900.7)	56.7 (1.5)	Neftekumsk, Burgun-Majary (Kurgan)
*C. album* (wetland) stems	67,379.2 (3061.4)	54,135.6 (2628)	50,713 (2629.2)	40,006.6 (965.6)	59.5 (3.1)	Mikhailovsk and Stavropol

## Data Availability

The datasets generated during the current study have been submitted to NCBI Sequence Reads Archive (SRA) with accession number PRJNA1306194.

## Supplementary Materials

The following supporting information can be downloaded at: https://www.mdpi.com/article/10.3390/microorganisms14102273/s1, Table S1: Meteorological data of season 2023 for the city Neftekumsk, Table S2: Meteorological data of season 2023 for village of Burgun-Majary, Table S3: Meteorological data of season 2023 for the cities Mikhailovsk and Stavropol, Table S4: Taxonomic affiliation of the identified microorganisms, Table S5: Pairwise PERMANOVA results; Figure S1: Functional prediction of bacterial communities based on FAPROTAX.
