# Supplementary material for: Analysis of Microbial Communities Associated with Drought-Resistant Plants Using 16S rRNA Sequencing
Source: Microorganisms. 2026 Oct 6;14(10):2273. doi: 10.3390/microorganisms14102273 (PMC13707488; doi:10.3390/microorganisms14102273)
Supplement: Supplementary file 1 [file microorganisms-14-02273-s001.zip › Figure S1.pdf]

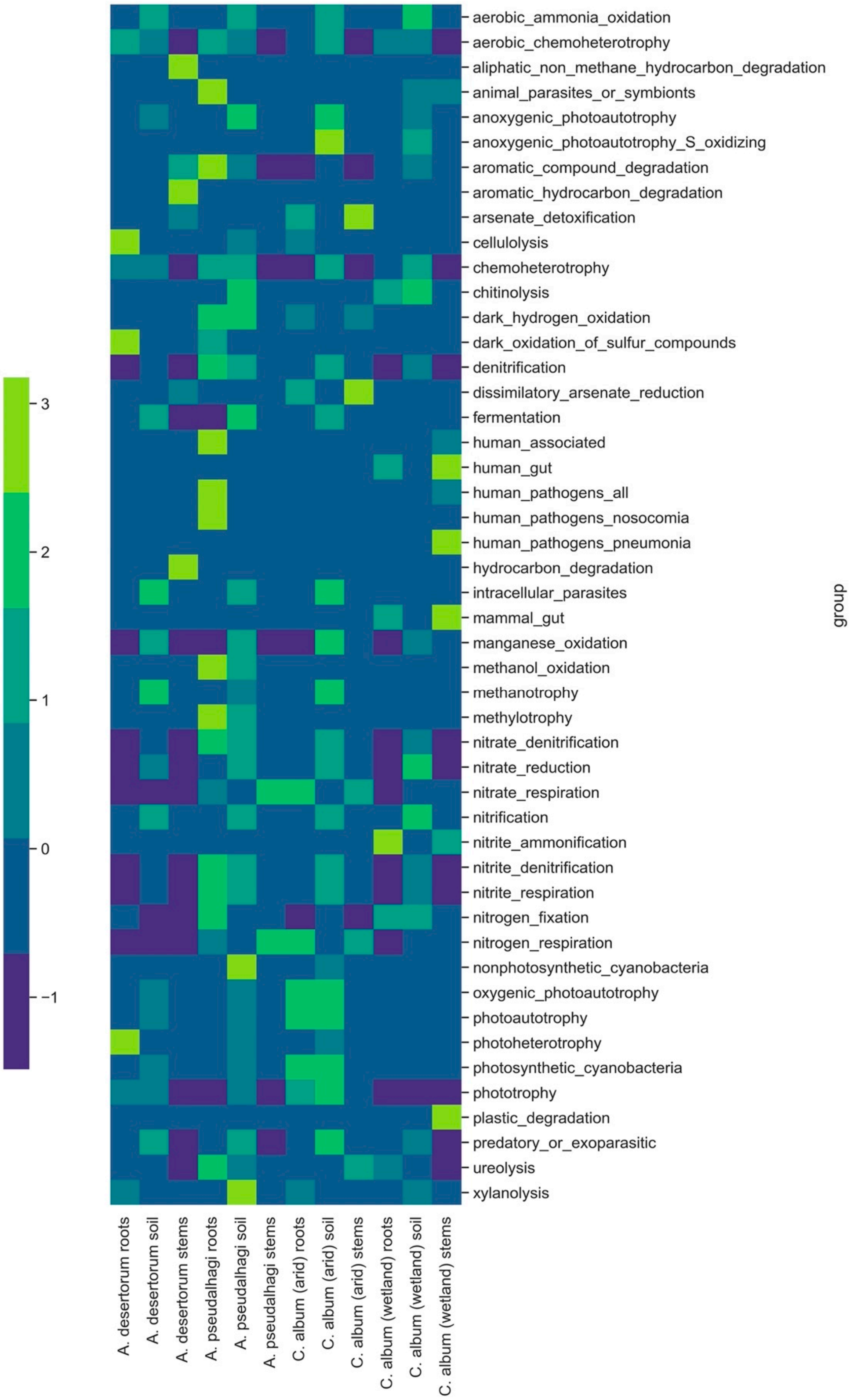

Figure S1. Functional prediction of bacterial communities based on FAPROTAX. The abundance values were z-scored.
