# Supplementary material for: Analysis of Microbial Communities Associated with Drought-Resistant Plants Using 16S rRNA Sequencing
Source: Microorganisms. 2026 Oct 6;14(10):2273. doi: 10.3390/microorganisms14102273 (PMC13707488; doi:10.3390/microorganisms14102273)
Supplement: Supplementary file 1 [file microorganisms-14-02273-s001.zip › Tables S1-S3.pdf]

Table S1. Meteorological data of season 2023 for the city Neftekumsk
(according to the Roschino weather station of the Neftekumsk district of the Stavropol Territory)

Month	01	02	03	04	05	06	07	08	09	10	11	12
Temperature	1.07	1.23	7.87	12.17	17.9	23.8	26.57	28	20	13	9.07	2.93
Precipitation	0	13.5	4.6	27.9	46.6	17.8	59.0	0.5	33.2	12.2	30.7	51.3

Table S2. Meteorological data of season 2023 for village of Burgun-Majary
(according to the Zelenokumsk weather station of the Zelenokumsk district of the Stavropol Territory)

Month	01	02	03	04	05	06	07	08	09	10	11	12
Temperature	0.27	1.23	7.77	11.87	16.6	22.27	25.17	27.07	19.37	12.6	8.17	2.2
Precipitation	0.6	24.0	15.4	35.8	66.0	100.0	95.0	21.0	6.3	15.0	59.8	32.2

Table S3. Meteorological data of season 2023 for the cities Mikhailovsk and Stavropol
(according to the Stavropol weather station of the Stavropol Territory)

Month	01	02	03	04	05	06	07	08	09	10	11	12
Temperature	0.5	1	5.5	11	15.5	20.5	23	24	18.5	12	6	1.5
Precipitation	7.4	82.3	57	18.1	92.5	60.7	28	11.5	15	41.2	38	22.4
